# REaCT-5G: a randomized trial of bone pain with 5-day filgrastim vs pegfilgrastim for neutropenia in breast cancer

**DOI:** 10.1093/jncics/pkaf081

**Published:** 2025-08-14

**Authors:** Terry L Ng, Peter Greenstreet, Carol Stober, Stuart Nicholls, Jennifer Shamess, Natalie Mills, Mohammed Ibrahim, Marie-France Savard, Moira Rushton, Arif Awan, Sandeep Sehdev, John Hilton, Xinni Song, Parvaneh Fallah, Nasser Alqahtani, Daniel Davoudpour, Kelly Daigle, Fiona MacDonald, Lisa Vandermeer, Monica Taljaard, Mark Clemons

**Affiliations:** Division of Medical Oncology, Department of Medicine, University of Ottawa, Ottawa, ON, Canada; Cancer Research Program, Ottawa Hospital Research Institute, Ottawa, ON, Canada; The Ottawa Hospital Cancer Centre, Ottawa, ON, Canada; Ottawa Methods Centre, Ottawa Hospital Research Institute, Ottawa, ON, Canada; Cancer Research Program, Ottawa Hospital Research Institute, Ottawa, ON, Canada; Methodological and Implementation Research Program, Ottawa Hospital Research Institute, Ottawa, ON, Canada; Office for Patient Engagement in Research Activity, Ottawa Hospital Research Institute, Ottawa, ON, Canada; Champlain Regional Cancer Care Patient and Family Advisory Council, Ottawa, ON, Canada; St Joseph Family Medical Clinic, Ottawa, ON, Canada; Department of Medical Oncology, Northern Ontario School of Medicine University, Thunder Bay, ON, Canada; Thunder Bay Regional Health Sciences Centre, Thunder Bay, ON, Canada; Division of Medical Oncology, Department of Medicine, University of Ottawa, Ottawa, ON, Canada; Cancer Research Program, Ottawa Hospital Research Institute, Ottawa, ON, Canada; The Ottawa Hospital Cancer Centre, Ottawa, ON, Canada; Division of Medical Oncology, Department of Medicine, University of Ottawa, Ottawa, ON, Canada; Cancer Research Program, Ottawa Hospital Research Institute, Ottawa, ON, Canada; The Ottawa Hospital Cancer Centre, Ottawa, ON, Canada; Division of Medical Oncology, Department of Medicine, University of Ottawa, Ottawa, ON, Canada; Cancer Research Program, Ottawa Hospital Research Institute, Ottawa, ON, Canada; The Ottawa Hospital Cancer Centre, Ottawa, ON, Canada; Division of Medical Oncology, Department of Medicine, University of Ottawa, Ottawa, ON, Canada; Cancer Research Program, Ottawa Hospital Research Institute, Ottawa, ON, Canada; The Ottawa Hospital Cancer Centre, Ottawa, ON, Canada; Division of Medical Oncology, Department of Medicine, University of Ottawa, Ottawa, ON, Canada; Cancer Research Program, Ottawa Hospital Research Institute, Ottawa, ON, Canada; The Ottawa Hospital Cancer Centre, Ottawa, ON, Canada; Division of Medical Oncology, Department of Medicine, University of Ottawa, Ottawa, ON, Canada; Cancer Research Program, Ottawa Hospital Research Institute, Ottawa, ON, Canada; The Ottawa Hospital Cancer Centre, Ottawa, ON, Canada; Gerald Bronfman Department of Oncology, McGill University, Montreal, Quebec, Canada; Division of Medical Oncology, Department of Medicine, University of Ottawa, Ottawa, ON, Canada; Department of Medicine, King Abdulaziz Hospital, Alhasa, Saudi Arabia; Thunder Bay Regional Health Sciences Centre, Thunder Bay, ON, Canada; The Ottawa Hospital Cancer Centre, Ottawa, ON, Canada; The Ottawa Hospital Cancer Centre, Ottawa, ON, Canada; Cancer Research Program, Ottawa Hospital Research Institute, Ottawa, ON, Canada; Ottawa Methods Centre, Ottawa Hospital Research Institute, Ottawa, ON, Canada; Methodological and Implementation Research Program, Ottawa Hospital Research Institute, Ottawa, ON, Canada; Division of Medical Oncology, Department of Medicine, University of Ottawa, Ottawa, ON, Canada; Cancer Research Program, Ottawa Hospital Research Institute, Ottawa, ON, Canada; The Ottawa Hospital Cancer Centre, Ottawa, ON, Canada

## Abstract

**Background:**

Granulocyte colony-stimulating factors (G-CSFs), including filgrastim and pegfilgrastim, are associated with bone pain, potentially impacting treatment adherence. This study hypothesized that a 5-day regimen of filgrastim would result in less bone pain than single-dose pegfilgrastim in patients receiving chemotherapy for early breast cancer.

**Methods:**

In this multicenter, open-label, randomized controlled trial, patients requiring prophylactic G-CSF during chemotherapy were randomly assigned 1:1 to receive either 5-day filgrastim or pegfilgrastim. The primary outcome was patient-reported bone pain, assessed as area under the curve of daily pain scores (0 = no pain to 10 = worst pain) over the first 5 days following G-CSF in cycle 1. Secondary outcomes included bone pain in cycles 2-4, febrile neutropenia, hospitalizations, chemotherapy delays, dose reductions, early discontinuations, chemotherapy-related deaths, health-related quality of life, and health-care resource utilization.

**Results:**

From June 2021 to March 2023, a total of 233 patients were randomly assigned, with 219 analyzed (110 filgrastim and 109 pegfilgrastim) after excluding those who withdrew before receiving treatment. Adjusting for stratification factors and prespecified baseline covariates using repeated measures linear regression, the mean area under the curve (0-40) for cycle 1 bone pain was 10.2 (11.2) for 5-day filgrastim and 10.2 (9.81) for pegfilgrastim, with an adjusted mean difference of 0.70 (95% confidence interval = 1.62 to 3.02; *P *= .556). Although no clinically significant differences were observed in most secondary outcomes, the 5-day filgrastim group exhibited a numerically higher incidence of febrile neutropenia (6.4% vs 0.9%, *P *= .065) and hospitalization (10.0% vs 3.7%, *P *= .106).

**Conclusion:**

There was no significant difference in bone pain between 5-day filgrastim and pegfilgrastim.

## Introduction

Granulocyte colony-stimulating factors (G-CSFs), such as filgrastim and its long-acting pegylated form, pegfilgrastim, are widely recommended for primary febrile neutropenia prophylaxis and for maintaining chemotherapy dose intensity in early breast cancer.^1-6^ However, G-CSF–induced bone pain can be severe, leading to emergency room visits, opioid use, and even early chemotherapy discontinuation, making it an important patient-centered outcome.^4^^,^^7^

Registry clinical trials have demonstrated that a single pegfilgrastim injection is noninferior to daily filgrastim in reducing febrile neutropenia incidence, duration, and related hospitalizations.^8^^,^^9^ Although these studies reported similar rates of bone pain between groups (any pain = 28%-42%; severe pain = <8%),^9-11^ 2 key issues remain. First, in these trials, filgrastim was administered for a median of 11 days, whereas subsequent studies suggest a shorter duration 5-day regimen of filgrastim is commonly used in clinical practice and is considered acceptable.^12^^,^^13^ Second, prior pegfilgrastim vs filgrastim studies assessing bone pain relied on clinician-reported outcomes, which often underestimate patient-reported experiences.^14-17^ Notably, a randomized trial evaluating naproxen for pegfilgrastim-related bone pain, using prospectively collected patient-reported outcome measures, found a substantially higher incidence of patient-reported pain (any pain = 71%; severe pain = 27%).^18^

Although pegfilgrastim offers more convenient dosing, some evidence suggests that it may cause more musculoskeletal pain than filgrastim.^19^ Additionally, a shorter 5-day filgrastim regimen, which is commonly used in real-world practice,^13^ may reduce G-CSF–induced bone pain because of its shorter duration of action. To address these gaps, we conducted a multicenter, randomized, open-label trial comparing the incidence and severity of bone pain between patients receiving 5-day filgrastim or a single dose of pegfilgrastim as primary febrile neutropenia prophylaxis during neoadjuvant or adjuvant chemotherapy for early breast cancer (NCT04781959), hypothesizing that 5-day filgrastim would cause less bone pain.

## Methods

### Study design

This multicenter, open-label, randomized controlled trial enrolled adults (aged 18 years and older) with early breast cancer who required primary febrile neutropenia prophylaxis with G-CSF during neoadjuvant or adjuvant chemotherapy per physician discretion. Participants were enrolled at 2 university cancer centers where they provided oral consent using an integrated verbal consent model.^20^

Patients were randomly assigned in a 1:1 ratio using stratified permuted blocks to receive either 5-day filgrastim at 300 μg (if <90 kg body weight) or 480 μg (if ≥90 kg body weight) or a single 6 mg injection of pegfilgrastim, administered 24-72 hours postchemotherapy. Random assignment was stratified by cancer center and chemotherapy regimen (taxane-based vs anthracycline-based chemotherapy in the first 4 cycles). Medication for musculoskeletal pain including antihistamines (eg, loratadine) were allowed. The study was approved by the Ontario Cancer research ethics board and registered on clinical trials.gov (NCT04781959).

### Outcomes

The primary outcome was patient-reported bone pain, assessed using the visual analogue scale (0 = no pain, 10 = worst pain) from days 1 to 5 post–G-CSF during cycle 1. Pain severity was quantified by the area under the curve (AUC), with a maximum score of 40 if a score of 10 was reported on all 5 days.^21^

Secondary outcomes included bone pain during cycles 2-4, incidence of febrile neutropenia (fever ≥ 38.3°C or 2 readings ≥ 38.0°C for ≥2 hours with an absolute neutrophil count less than 0.5 × 10^9^/L),^22^ treatment-related hospitalization (both febrile neutropenia and nonfebrile neutropenia related), chemotherapy dose delays, dose reductions, premature chemotherapy discontinuation, and chemotherapy-related mortality (death from chemotherapy initiation to 1 month posttreatment) and were assessed after each cycle of chemotherapy. Health-related quality of life (HRQOL) was assessed using the EuroQol (EQ)-5D-5L and the functional interference subscale of the European Organization for Research and Treatment of Cancer (EORTC) QOL questionnaire—bone metastases module (EORTC-QLQ-BM22). Health-care resource utilization was assessed based on the number of emergency room visits, unplanned clinic visits, and patient support line interactions recorded during the study. Methods of bone pain assessment were extensively discussed, and the use of the BM22 functional interference subscale and 5D-5L were endorsed by our patient partners (J.S. and N.M.).

### Study procedures

Participants recorded daily visual analogue scale bone pain scores (most severe bone pain experienced in the past 24 hours) for up to 8 days, starting 24 hours after G-CSF administration across cycles 1-4.^21^ The modified EORTC-QLQ-BM22 (functional interference) and EQ-5D-5L (overall health and cost utility) were completed at baseline and on day 8 post–G-CSF during each cycle. Follow-up continued until 1 month after chemotherapy completion to assess chemotherapy-related mortality.

### Random assignment

An independent statistician at the Ottawa Hospital Research Institute generated the allocation sequence using stratified permuted blocks of varying lengths. After obtaining informed consent and confirming eligibility, research coordinators accessed a secure, password-protected website to obtain the next treatment assignment. The treatment allocation was concealed from patients, investigators, and study staff until the time of random assignment allocation, after which the study coordinator would notify the patient and treating physician of the allocated intervention. Blinding was not feasible given the nature of the study intervention (5 injections vs 1 injection).

### Sample size

A sample size of 220 patients (110 per arm) was determined to detect a minimum clinically important difference of 2.0 (8.7) in AUC bone pain scores (based on Kirshner et al.^21^) with 80% power. This calculation employed a 2-sided test at the 5% level and adjusted for correlation with an estimated baseline pain score of 0.8. Allowing for a 5% attrition rate, the target enrollment was increased to 232 (116 per arm).

### Analysis plan

Baseline characteristics and categorical outcomes were summarized as frequencies and proportions, whereas continuous outcomes were reported as means and standard deviations.

The primary outcome, the AUC for bone pain during days 1-5 post–G-CSF in cycle 1, was calculated for each patient across chemotherapy cycles. To enhance statistical power and efficiency, stratification factors—cancer center and chemotherapy regimen—were included as fixed covariates along with prespecified baseline prognostic factors for pain (age, pain at baseline, and use of pain medications at baseline), in accordance with recommendations for stratified allocation.^23-26^ AUC measures from the first 4 chemotherapy cycles were then analyzed using a repeated measures linear regression model, incorporating terms for treatment, cycle, and cycle-by-treatment interaction. The model, estimated using restricted maximum likelihood with an unstructured covariance matrix, accounted for within-patient correlations across repeated measures and provided adjusted mean differences between treatment arms with 95% confidence intervals (CIs) for the prespecified primary outcome at cycle 1, as well as for comparisons across cycles 2-4.

Secondary outcomes measured as continuous variables (eg, EQ-5D-5L health score) were analyzed as described for the primary outcome. Secondary outcomes measured as binary variables (eg, incidence of febrile neutropenia and hospitalization) were analyzed using Fisher exact test because of the low number of events. Count data (eg, number of emergency room visits) were analyzed using unadjusted Poisson regression.

Subgroup analyses for the primary outcome were performed based on the stratification variables: center and chemotherapy regimen. These analyses were conducted using data from cycle 1 and included the interaction between the treatment and subgroup variables in the models.

## Results

### Study population

**Figure 1.**
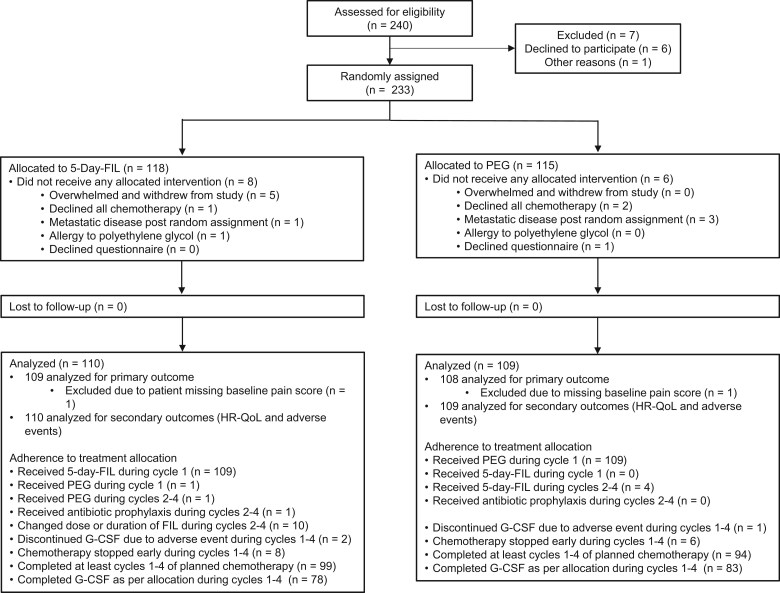
CONSORT diagram. Abbreviations: FIL = filgrastim; G-CSFs = granulocyte colony-stimulating factors; PEG = pegfilgrastim.

Between June 2021 and March 2023, a total of 233 eligible patients were randomly assigned. Among these, 14 (6.0%) patients withdrew before receiving any study intervention and were excluded from all analyses, as they provided no bone pain data for any treatment cycle. The final analysis included 219 patients (5-day filgrastim: *n* = 110; pegfilgrastim: *n* = 109). Only 4 patients in the 5-day filgrastim group received the 480 μg dose. In addition, adherence to treatment allocation during cycles 1-4 of chemotherapy is summarized (Figure 1).

### Baseline characteristics

Baseline characteristics (Table 1) were well balanced between groups, with similar proportions of patients receiving anthracycline-based and taxane-based chemotherapy and pain medications at baseline. Mean baseline pain scores (out of 10), prior to starting chemotherapy and G-CSF, were 1.05 (1.75) in the filgrastim group and 1.15 (1.81) in the pegfilgrastim group.

**Table 1.** Baseline patient characteristics before study enrollment.

Characteristics	5-day filgrastim (*n* = 110)	Pegfilgrastim (*n* = 109)
Age, mean (SD), y	55.7 (12.6)	55.6 (12.5)
Sex, No. (%)		
Male	2 (1.8)	0 (0)
Female	108 (98.2)	109 (100)
Chemotherapy, No. (%)		
Anthracycline-based	49 (44.5)	48 (44.0)
Taxane-based	61 (55.5)	61 (56.0)
Trastuzumab, No. (%)		
Yes	26 (23.6)	40 (36.7)
No	84 (83.0)	69 (63.3)
Baseline pain score, mean (SD)	1.05 (1.75)	1.15 (1.81)
Pain medications at baseline, No. (%)		
Opioid	4 (3.6)	3 (2.8)
Acetaminophen	22 (20.0)	21 (19.3)
Nonsteroidal anti-inflammatory drugs	11 (10.0)	11 (10.1)
Other^a^	12 (10.9)	7 (6.4)

a “Other” medications included antihistamines (loratadine and nonloratidine) and less common agents including gabapentin, dexamethasone, cannabinoid and/or tetrahydrocannabinol oil, baclofen, and cyclobenzaprine.

### Primary outcome

As specified in the study protocol, after adjusting for stratification factors and prespecified prognostic covariates, the adjusted mean difference in AUC bone pain scores during cycle 1 was 0.70 (95% CI = −1.62 to 3.02; *P *= .556) indicating no statistically significant difference between 5-day filgrastim and pegfilgrastim (Table 2). One patient from each of the 5-day filgrastim and pegfilgrastim groups was excluded because of missing baseline pain scores required for the primary outcome analysis model. There were also no differences observed in patient-reported daily bone pain scores across cycles 2-4.

**Table 2.** Analysis of the primary and secondary outcomes over cycles 1-4.

Bone pain and secondary quality of life (QoL) endpoints	5-day filgrastim, mean (SD) (*n* = 109)	Pegfilgrastim, mean (SD) (*n* = 108)	Adjusted mean difference^a^ (95% CI)	*P* ^b^	*P*_interaction_ ^c^
Bone pain area under the curve					.483
Cycle 1, primary endpoint	10.7 (11.2)	10.2 (9.81)	0.70 (−1.62 to 3.02)	.556	
Cycle 2	8.41 (9.55)	7.51 (8.32)	1.04 (−1.28 to 3.36)	.380	
Cycle 3	8.78 (9.70)	8.25 (8.90)	0.71 (−1.63 to 3.05)	.550	
Cycle 4	8.42 (9.84)	8.87 (9.93)	−0.42 (−2.78 to 1.94)	.727	
EuroQol-5D-5L					.595
Cycle 1	76.0 (18.6)	79.1 (17.4)	−2.31 (−6.24 to 1.62)	.249	
Cycle 2	76.9 (15.4)	78.3 (15.0)	−0.52 (−4.45 to 3.41)	.795	
Cycle 3	74.0 (17.1)	77.2 (15.4)	−2.32 (−6.29 to 1.65)	.252	
Cycle 4	73.9 (17.4)	75.1 (16.9)	−0.20 (−4.24 to 3.85)	.925	
European Organization for Research and Treatment of Cancer-Quality of Life-BM22					.533
Cycle 1	1.81 (0.699)	1.89 (0.673)	−0.086 (−0.235 to 0.062)	.255	
Cycle 2	1.66 (0.636)	1.64 (0.560)	0.007 (−0.142 to 0.155)	.932	
Cycle 3	1.67 (0.670)	1.68 (0.533)	0.036 (−0.186 to 0.114)	.640	
Cycle 4	1.74 (0.730)	1.71 (0.631)	0.012 (−0.164 to 0.140)	.880	

a Results were obtained from repeated measures linear regression analyses adjusting for prespecified baseline covariates.

b *P*-value of the between-arm contrast at each cycle.

c Significance of the group-by-cycle interaction.

### Secondary outcomes

Table 3 summarizes the secondary outcomes measured as binary variables (ie, adverse event outcomes). Over the full study period (up to 8 cycles of chemotherapy), the incidence of febrile neutropenia was 6.4% vs 0.9% (*P *= .065) and hospitalization was 10.0% vs 3.7% (*P *= .106) in the 5-day filgrastim and pegfilgrastim groups, respectively. Similar trends were observed when restricting the analysis to cycles 1-4, a key consideration given that not all participants received more than 4 chemotherapy cycles. No statistically significant differences were found in chemotherapy delay, dose reduction, or premature discontinuation. One death occurred in the 5-day filgrastim group, though it was not attributed to chemotherapy. The participant discontinued treatment after cycle 2 and experienced progressive clinical decline until death. No deaths were reported during cycles 1-4.

**Table 3.** Fisher exact analyses of the incidence of adverse events.

Chemotherapy-related adverse events, No. (%)	5-day filgrastim (*n* = 110)	Pegfilgrastim (*n* = 109)	*P*
Over the full trial			
Febrile neutropenia	7 (6.36%)	1 (0.92%)	.065
Hospitalization (febrile neutropenia and nonfebrile neutropenia related)	11 (10.0%)	4 (3.67%)	.106
Chemotherapy delay	22 (20.0%)	21 (19.3%)	1.000
Dose reduction	36 (32.7%)	35 (32.1%)	1.000
Premature chemotherapy discontinuation	22 (20.0%)	24 (22.0%)	.886
Chemotherapy-related deaths	1 (0.91%)	0 (0%)	1.000
Over cycles 1-4			
Febrile neutropenia	6 (5.45%)	1 (0.92%)	.119
Hospitalization (febrile neutropenia and nonfebrile neutropenia related)	11 (10.0%)	3 (2.75%)	.0497
Chemotherapy delay	14 (12.7%)	13 (11.9%)	1.000
Dose reduction	17 (15.5%)	17 (15.6%)	.989
Premature chemotherapy discontinuation	9 (8.18%)	6 (5.50%)	.594
Chemotherapy-related deaths	0 (0%)	0 (0%)	1.000

HRQOL (Table 2) showed no statistically significant differences between treatment groups in EQ-5D-5L overall health or EORTC-QLQ-BM-22 functional interference subscale scores.

Table 4 presents the health-care utilization analysis. Although the number of cancer center patient support line interactions and emergency room visits was similar, unplanned clinic visits were statistically significantly higher in the 5-day filgrastim group over all treatment cycles (13 vs 4; *P *= .041) and over cycles 1-4 (10 vs 2; *P *= .039). However, an exploratory analysis restricted to health-care utilization events related to fever and pain management found that only 2 patients in each treatment group had unplanned visits for pain, and none were for fever (Table S1).

**Table 4.** Poisson regression analyses of health-care utilization (secondary outcomes).

Unplanned healthcare resource utilization	5-day filgrastim	Pegfilgrastim	Ratio of events (95% CI)	*P*
Over all cycles				
No. of emergency room visits				
Per patient, mean (SD)	0.255 (0.582)	0.266 (0.588)	0.957	.867
Total No. of visits	28	29	(0.569 to 1.608)	
Unplanned clinic visits				.041
Per patient, mean (SD)	0.118 (0.444)	0.037 (0.189)	3.220	
Total No. of visits	13	4	(1.050 to 9.877)	
No. of calls				.204
Per patient, mean (SD)	0.572 (1.000)	0.450 (0.822)	1.274	
Total No. of visits	63	49	(0.877 to 1.851)	
Over cycles 1-4				
No. of emergency room visits				.730
Per patient, mean (SD)	0.173 (0.466)	0.193 (0.518)	0.897	
Total No. of visits	19	21	(0.482 to 1.668)	
Unplanned clinic visits				.039
Per patient, mean (SD)	0.091 (0.372)	0.018 (0.135)	4.954	
Total No. of visits	10	2	(4.955 to 22.61)	
No. of calls				.207
Per patient, mean (SD)	0.373 (0.800)	0.275 (0.665)	1.354	
Total No. of visits	41	30	(0.846 to 2.169)	

Abbreviation: CI = confidence interval.

### Subgroup analyses

**Figure 2.**
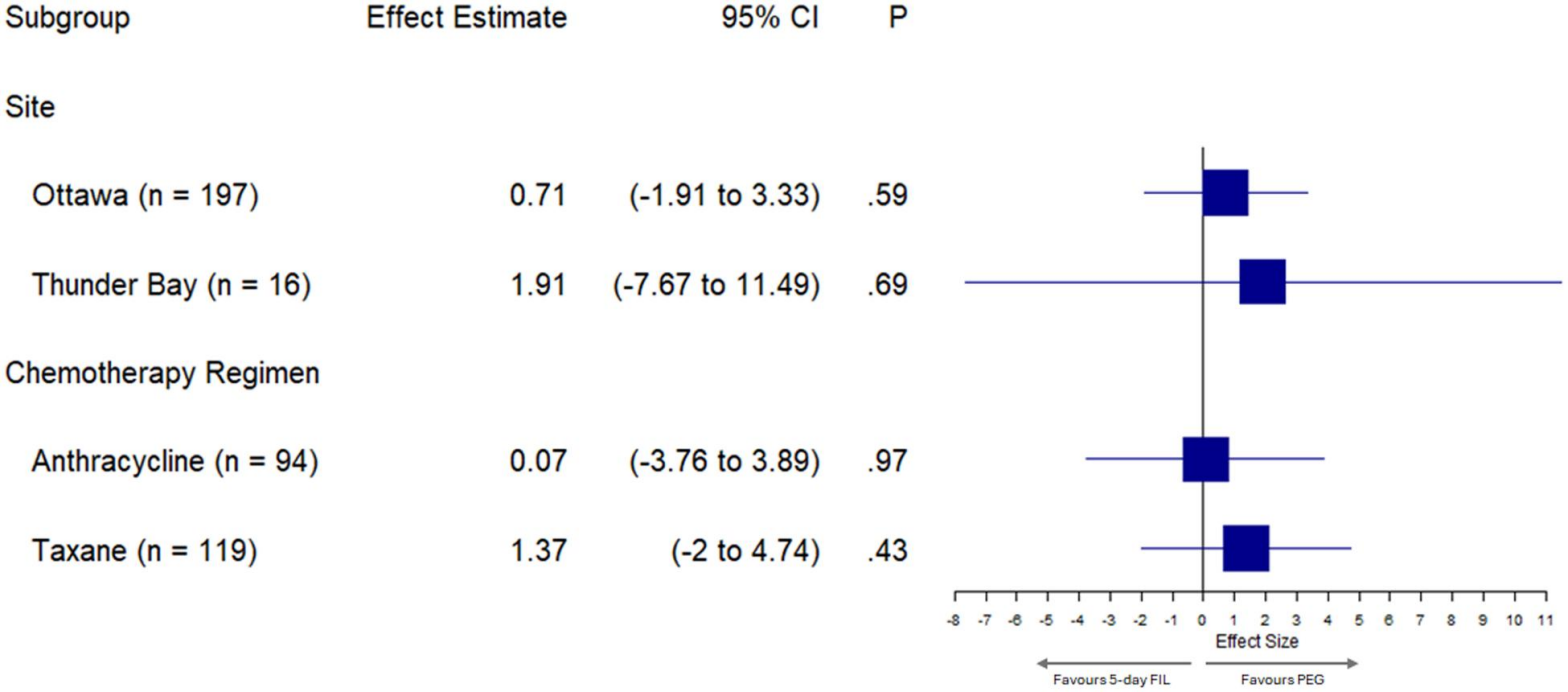
Preplanned analysis of bone pain based on stratified subgroups. Abbreviations: CI = confidence interval; FIL = filgrastim; PEG = pegfilgrastim.

Preplanned subgroup analyses (Figure 2), which included only patients with complete baseline and cycle 1 bone pain data, showed no statistically significant differences in bone pain for the 5-day filgrastim and pegfilgrastim groups by site (Ottawa: *P *= .593; Thunder Bay: *P *= .695) or by chemotherapy type (anthracycline: *P *= .973; taxane: *P *= .426). As expected, mean bone pain scores were higher in the taxane chemotherapy subgroup (Table S2).

Antihistamines, a potential strategy to mitigate G-CSF–associated bone pain, given at baseline and during chemotherapy cycles 1-4 (Table S3), and the rates of febrile neutropenia and hospitalizations in participant subgroups receiving dose-dense vs standard chemotherapy (Table S4), have been summarized descriptively. A post hoc subgroup analysis evaluating bone pain during cycle 1 based on the use of dose-dense (*P *= .994) and standard chemotherapy (*P *= .329) did not identify statistically significant differences (Table S5 and Figure S1).

## Discussion

G-CSF is a cornerstone of supportive care for patients undergoing chemotherapy, particularly those at high risk (≥20%) of febrile neutropenia^22^; these include most patients receiving chemotherapy for early breast cancer. Although prior randomized controlled trials (RCTs) compared pegfilgrastim and filgrastim, those studies administered filgrastim for a median of 11 days—longer than the 5-7 days commonly used in clinical practice.^9^^,^^10^ Additionally, bone pain assessments in prior studies relied on observer or physician reports rather than patient-reported outcomes. To address these gaps, **RE**thinking **C**linical **T**rals (REaCT-5G) was designed to compare 5-day filgrastim, a regional standard, with pegfilgrastim, focusing on patient-reported bone pain.

In our study, no statistically or clinically significant difference in patient-reported bone pain was observed between the 5-day filgrastim and pegfilgrastim groups. Similarly, no statistically significant differences were observed in HRQOL including functional interference (EORTC-QLQ-BM22) and overall health scores (EQ-5D-5L). Although most secondary outcomes were comparable, the 5-day filgrastim group had a numerically higher incidence of febrile neutropenia and hospitalization and unplanned clinic visits. However, the study was not powered to detect significant between-group differences for these secondary outcomes.^12^ As per our analysis plan, a 2-sided alpha level of .05 was used instead of a 1-sided .05 level, which may have underestimated potentially important differences in adverse events. Therefore, these results should be interpreted with caution.

Several factors may account for the observed differences in febrile neutropenia and hospitalization. First, the shorter 5-day filgrastim regimen may have contributed to higher febrile neutropenia risk compared with the 7-10 or more days regimens commonly used in practice. Second, unlike prior RCTs that employed weight-based dosing, this study used a fixed dosing strategy (300 μg/day or 480 μg/day if ≥ 90 kg) as per the regional standard practice, which may have influenced outcomes. Third, unlike early RCTs, which administered G-CSF 24 hours postchemotherapy, this pragmatic study allowed for administration within 24-72 hours, a common real-world practice that may impact neutropenia duration and clinical outcomes.^27^

This study is among the few RCTs to use patient-reported outcomes as a primary outcome to assess G-CSF–induced bone pain, comparing pegfilgrastim and 5-day filgrastim, addressing prior concerns that pegfilgrastim may be associated with increased bone pain. The results do not indicate statistically significantly greater patient-reported bone pain with pegfilgrastim compared with a shorter duration (5 days) of filgrastim. Although the study was powered to detect a small but clinically meaningful difference in bone pain (a 2-point change on a 40-point AUC scale), a larger-than-expected standard deviation resulted in wider confidence intervals than anticipated.

To mitigate bias, random assignment was stratified by cancer center and chemotherapy regimen, ensuring a balanced proportion of patients receiving taxane-based chemotherapy, which is associated with chemotherapy-induced musculoskeletal pain.^28^ Although sex was a prespecified covariate, it was not included in the final analysis model, as only 2 participants were male.

The study has limitations. The inclusion of only 2 Canadian cancer centers limits the generalizability of findings to broader populations with varying health-care infrastructures, socioeconomic backgrounds, racial and ethnic identities, and cultural factors. Additionally, the study was not powered to assess febrile neutropenia and hospitalization as primary endpoints. The use of fixed-dose G-CSF rather than weight-based dosing, as used in registration trials, may have affected bone pain and febrile neutropenia incidence but reflects real-world prescribing patterns. Allowing flexibility in the timing of G-CSF administration (up to 72 hours postchemotherapy) may have influenced outcomes compared with more standardized administration times. Last, a small proportion (6%) of patients withdrew before starting any study intervention and had to be excluded from the analysis, but our sample size was increased to account for an anticipated 5% attrition rate. Postrandom assignment exclusions may introduce bias if exclusions are differential between study arms; however, we have no reason to suspect that postrandom assignment exclusions skewed interpretation of our results, as the proportion of excluded patients was small.

Pegfilgrastim remains an effective and convenient option, offering a single-dose alternative to filgrastim, which is typically administered over 5-10 days. However, pegfilgrastim ($1375 CAD per 6 mg dose) is significantly more expensive than shorter duration filgrastim regimens ($138.54 per 300 μg dose × 5 days = $692.7; $221.66 per 480 μg dose × 5 days = $1108.3).^29^^,^^30^ Notably, all but 4 patients in our study received 300 μg/day of filgrastim. If cost and access were not limiting factors, pegfilgrastim could be the preferred option because of its convenience, potential for improved adherence, and comparable efficacy. Patient preference and cost utility analyses were conducted as part of this study and will be reported separately. Shorter duration filgrastim remains a viable alternative, particularly in resource-limited settings or where financial constraints influence treatment decisions and health policy.

These findings support the continued use of pegfilgrastim in resource-rich health systems, however, further research is needed to optimize neutropenia management.^31^ For instance, although ciprofloxacin is associated with higher rates of febrile neutropenia than G-CSF in early breast cancer, the incremental cost-effectiveness ratio of G-CSF vs ciprofloxacin was estimated at $1.76 million CAD per quality-adjusted life-year gained, much higher than the commonly used willingness-to-pay value of $50 000 CAD per quality-adjusted life-year.^32^ Larger studies in more diverse patient populations are warranted to enhance the generalizability of these findings and guide clinical decision making.

## Supplementary Material

pkaf081_Supplementary_Data

## Data Availability

All data are incorporated into the article and its online supplementary material. Patient-level data for future research collaborations contain personal health identifying information and would require separate research ethics board approval.
