# Correction to: REaCT-5G: a randomized trial of bone pain with 5-day filgrastim vs pegfilgrastim for neutropenia in breast cancer

**DOI:** 10.1093/jncics/pkag016

**Published:** 2026-03-11

This is a correction to Terry L Ng, Peter Greenstreet, Carol Stober, Stuart Nicholls, Jennifer Shamess, Natalie Mills, Mohammed Ibrahim, Marie-France Savard, Moira Rushton, Arif Awan, Sandeep Sehdev, John Hilton, Xinni Song, Parvaneh Fallah, Nasser Alqahtani, Daniel Davoudpour, Kelly Daigle, Fiona MacDonald, Lisa Vandermeer, Monica Taljaard, Mark Clemons, REaCT-5G: a randomized trial of bone pain with 5-day filgrastim vs pegfilgrastim for neutropenia in breast cancer, *JNCI Cancer Spectrum*, Volume 9, Issue 5, October 2025, pkaf081, https://doi.org/10.1093/jncics/pkaf081

Shortly after publication, a reader contacted the journal about data discrepancies between the Abstract and Table 2. The journal contacted all co-authors in line with COPE guidance, and after reviewing the concern, the authors confirmed that two corrections were needed in the Results section of the Abstract. Specifically, the second sentence has been changed from “Adjusting for stratification factors and prespecified baseline covariates using repeated measures linear regression, the mean area under the curve (0-40) for cycle 1 bone pain was 10.2 (11.2) for 5-day filgrastim and 10.2 (9.81) for pegfilgrastim, with an adjusted mean difference of 0.70 (95% confidence interval = 1.62 to 3.02; *P *= .556).” to “Adjusting for stratification factors and prespecified baseline covariates using repeated measures linear regression, the mean area under the curve (0-40) for cycle 1 bone pain was 10.7 (11.2) for 5-day filgrastim and 10.2 (9.81) for pegfilgrastim, with an adjusted mean difference of 0.70 (95% confidence interval = –1.62 to 3.02; *P* = .556).”

The authors regret these errors, which have been corrected online.

