# Supplementary material for: REaCT-5G: a randomized trial of bone pain with 5-day filgrastim vs pegfilgrastim for neutropenia in breast cancer
Source: JNCI Cancer Spectr. 2025 Aug 14;9(5):pkaf081. doi: 10.1093/jncics/pkaf081 (PMC12471350; doi:10.1093/jncics/pkaf081)

Table S1. Exploratory analysis of the number of healthcare resource utilization events for fever and pain management

|  | | Emergency Room | Unplanned clinic visits | Phone calls |
| --- | --- | --- | --- | --- |
| FIL | Fever | 11 | 0 | 4 |
|  | Pain | 1 | 2 | 25 |
| PEG | Fever | 10 | 0 | 7 |
|  | Pain | 1 | 2 | 19 |

Table S2. Results from the prespecified subgroup analyses for the primary outcome

|  | 5 Days FIL  Mean (SD) | PEG  Mean (SD) | Adjusted Mean difference (95% CI) | Subgroup p-value | Interaction p-value |
| --- | --- | --- | --- | --- | --- |
| **Site** |  |  |  |  | 0.812 |
| Ottawa (N=198) | 11.3 (11.3) | 10.6 (9.94) | 0.711  (-1.91, 3.33) | 0.593 |  |
| Thunderbay (N=16) | 5.35 (8.85) | 3.58 (3.51) | 1.91  (-7.67, 11.5) | 0.695 |  |
| **Chemotherapy** |  |  |  |  | 0.617 |
| Anthracycline (N=94) | 5.13 (7.14) | 5.61 (6.70) | 0.0652  (-3.76, 3.89) | 0.973 |  |
| Taxane (N=120) | 15.3 (11.8) | 13.6 (10.3) | 1.37  (-2.00, 4.74) | 0.426 |  |

Table S3. Use of Antihistamine medications at baseline and during chemotherapy cycles 1-4.

|  | **5 Days FIL**  **N=110** | **PEG**  **N=109** |
| --- | --- | --- |
| **Antihistamine medications at baseline, n (%)**  **Loratidine**  **Non-loratidine**  **Both** | 3 (2.7%)  7 (6.4%)  0 (0%) | 1 (0.9%)  4 (3.7%)  0 (0%) |
| **Antihistamine medications during cycles 1-4, n (%)**  **Loratidine**  **Non-loratidine**  **Both** | 20 (18.2%)  19 (17.3%)  8 (7.3%) | 19 (17.4%)  14 (12.8%)  2 (1.8%) |

Table S4. Post-hoc subgroup analyses of the incidence of febrile neutropenia and hospitalization based on standard vs. dose-dense chemotherapy schedule.

|  | **5 Days FIL**  **N=110** | **PEG**  **N=109** |
| --- | --- | --- |
| **Chemotherapy regimen, n (%)**  **Standard**  **Dose-dense** | 65 (59.1%)  45 (40.9%) | 69 (63.3%)  40 (36.7%) |
| **Incidence of Febrile Neutropenia**  **Standard**  **Dose-dense** | 4 (6.15%)  2 (4.44%) | 0 (0 %)  1 (2.5%) |
| **Incidence of Hospitalization**  **Standard**  **Dose-dense** | 9 (13.8%)  2 (4.44%) | 2 (2.90%)  1 (2.5%) |

Table S5. Post-hoc subgroup analyses for the primary outcome based on standard vs. dose-dense chemotherapy schedule.

|  | 5 Days FIL  Mean (SD) | PEG  Mean (SD) | Adjusted Mean difference (95% CI) | Subgroup p-value | Interaction p-value |
| --- | --- | --- | --- | --- | --- |
| **Chemotherapy** |  |  |  |  | 0.538 |
| Standard (N=130) | 14.7 (11.8) | 12.8 (10.2) | 1.61  (-1.64, 4.86) | 0.329 |  |
| Dose-dense (N=84) | 5.07 (7.23) | 5.75 (7.40) | -0.02  (-4.10, 4.07) | 0.994 |  |

Figure S1. Post-hoc subgroup analysis of cycle 1 bone pain based on standard vs. dose-dense chemotherapy schedule.


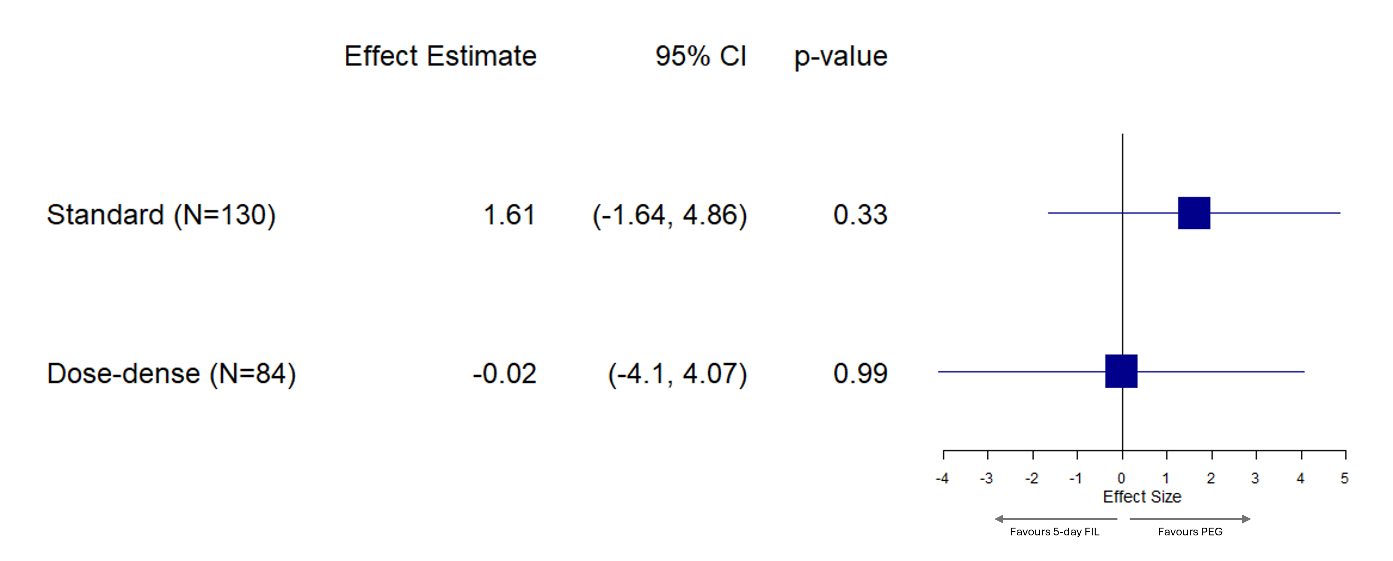

Supplement: pkaf081_Supplementary_Data [file pkaf081_supplementary_data.docx]
